# Tissue Doppler and strain imaging: anything left in the echo-lab?

**DOI:** 10.1186/1476-7120-6-54

**Published:** 2008-10-30

**Authors:** Rodolfo Citro, Eduardo Bossone, Bettina Kuersten, Giovanni Gregorio, Alessandro Salustri

**Affiliations:** 1 Department of UTIC-Cardiology, "San Luca" Hospital, Vallo della Lucania (SA), Italy; 2 Department of Cardiology, "Santa Maria dell'Olmo" Hospital, Cava dei Tirreni e Costa d'Amalfi (SA), Italy; 3 Department of Cardiology, Policlinico Luigi Di Liegro, Roma, Italy

## Abstract

Medline research indicates that an increasing number of manuscripts have been published in the last decade claiming, the feasibility and the potential clinical role of tissue Doppler and strain/strain rate imaging. However, despite this amount of scientific evidence, these technologies are still confined to dedicated, high-tech, research-oriented echocardiography laboratories. In this review we have critically evaluated these techniques, analysing their physical principles, the technical problems related to their current clinical application, and the future perspectives. Finally, this review explores the reasons why these technologies are still defined "new technologies" and the impact of their implementation on the current clinical activity of an echocardiography laboratory.

## Background

**Figure 1:**
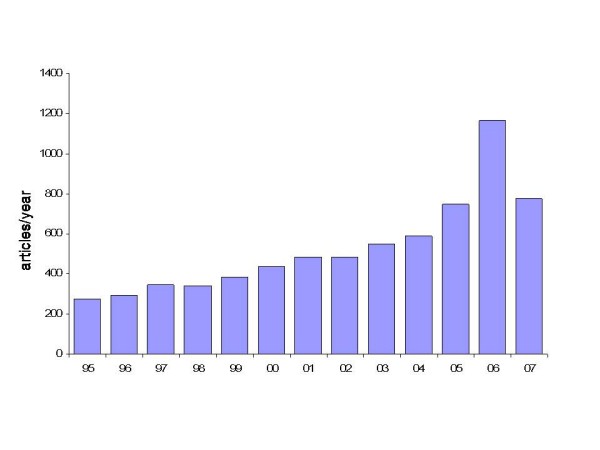
**Number of articles per year, reported on PubMed, entering the search filter "tissue Doppler" or "myocardial Doppler" in title and/or abstract.** Please, note the progressive increase in publications from the mid 90 s to the end of the year 2006.

In the past decade, numerous studies have been published addressing the feasibility and potential clinical applicability of tissue Doppler imaging (TDI) and its derived parameters strain and strain rate (SR) [1-7] (Figure 1). The data reported in these studies strongly support that the different methods are attractive in regards to their underlying theory as well as for the information revealed about cardiac function, that can be obtained by analyzing the motion of the diverse structural components of the heart (myocardial walls, valvular rings); at the same time all these methods strive to *eliminate everything subjective *in the assessment of an echocardiogram. Thus, a large volume of scientific evidence has been produced in favor of clinical use of these methods and various parameters have been proposed with the ambitious goal to contribute towards additional diagnostic value with respect to various pathologies. [8]. Despite this promising research, the parallel clinical diffusion of these methods never took place. To this day these methods are used only in a few centers and mainly in conjunction with research protocols; why have these published data not led to wider application in practice? Is this skepticism justified? Is the information obtained by TDI or SR 'redundant'? Do solid fields of clinical applications supported by these data already exist today or will they exist in the near future? In order to answer these questions, this review will first assess the various aspects of this technology from the physical principles to the technological implementation and then compile the information.

### Physical principles

**Figure 2:**
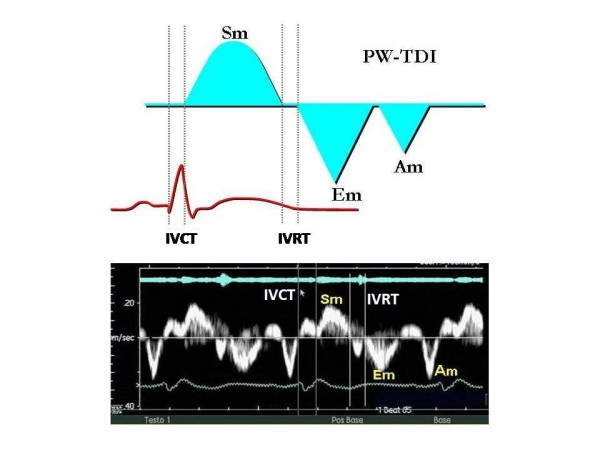
**Example of a normal PW-TDI spectral curve: note the immediate representation of the mechanical events in the various phases of the cardiac cycle.** It is possible to measure time intervals using the traces of the ECG and/or the phonocardiogram as reference (see the panel below). Sm = positive wave of systolic contraction; Em = negative early diastolic wave; Am = negative end-diastolic wave; IVCT = isovolumetric contraction time or precontraction; IVRT = isovolumetric relaxation time.

TDI, through proper modifications of the hardware and software of the ultrasound platform (elimination of the *high-pass filter *and adjustment of *gain*) allows the analysis of velocity signals having high amplitude and low frequency originating from tissue, which are usually not detected in traditional Doppler examination. In its various modalities (pulsed Doppler, 2D color mode and color M-mode), TDI renders possible *on-line *or *off-line*, the non invasive calculation of the time intervals and velocities of myocardial contraction and relaxation during the various phases of the cardiac cycle by using post-processing application software. (Figure 2). One of the primary reasons for the skepticism towards TDI lies in its very physical principles. Doppler echocardiography seems to be the ideal method for the examination of intracardiac and vascular flow that obey the laws of fluid dynamics and for the calculation of pressure gradients comparable to those obtained by invasive methods like cardiac catheterization. [8]. However, the motion of the myocardial walls is different, occurring in more directions and planes, and is influenced by the motion of the other organs and structures of the thoracic cage, following complex mechanical phenomena often not completely understood and for which there exists no reference method [10]. This problem is particularly evident when quantitative analysis of the myocardial function using TDI is applied to the study of segmental contraction abnormalities typical of ischemic cardiopathy.

**Figure 3:**
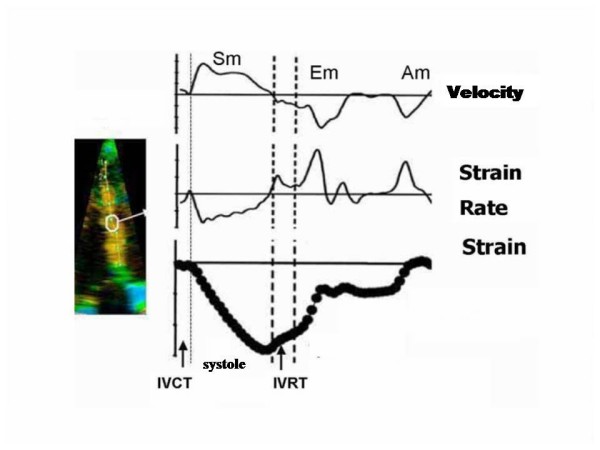
**New generation ultrasound platforms also allow to obtain simultaneous curves of velocity, strain rate and myocardial strain from a selected region of interest, for example the middle segment of the interventricular septum (note the white circle).** Other abbreviations as in figure 2. Modified from Sutherland GR et al [6].

In fact, if on one hand TDI has the merit of having brought back to mind very old concepts of physiopathology of the ischemic heart muscle, focusing the attention back on regional contraction phenomena, such as the delayed and reduced systolic shortening and the appearance of late systolic contraction, on the other hand, one must keep in mind that the measurement of regional myocardial velocities is not independent from the overall motion of the heart and suffers from tethering induced by collateral segments [3]. These limitations could potentially be overcome by strain imaging, which introduced the concept of myocardial deformation as a sensitive index of contractile capacity intrinsic to the myocardium [11] (Figure 3). However, strain is derived from the myocardial velocity gradient measured with TDI, from which the relative limitations in terms of angle-dependency are derived. Furthermore, the signal is strongly subjected to tedious noise problems (especially in the apical regions) which change the profiles of the spectral curve, making interpretation difficult, hardly reproducible and particularly arduous to reach interpretational consensus on, especially in the SR modality [4-6].

**Figure 4:**
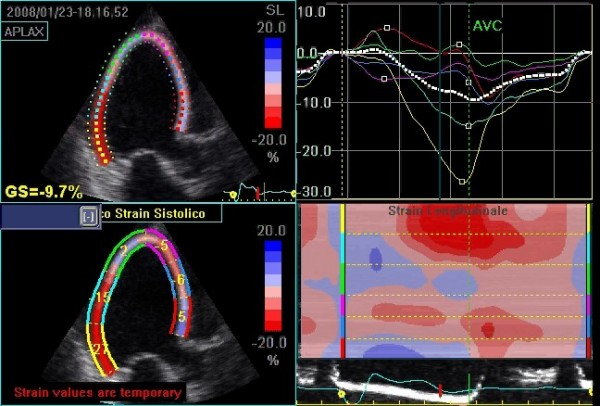
**Representation of 2D-strain (speckle tracking) of the left ventricle in the apical long axis view in a patient with systolic heart failure.** The upper right panel depicts the strain curves sampled in each of the analyzed myocardial segments. It is also possible to calculate a global strain index (GS), which in this case is clearly diminished (GS = 9,7%; see upper left panel). The lower left panel shows peak systolic strain for each segment while the lower right panel shows longitudinal strain processed according to a color map of curvilinear anatomical M-mode. From top to bottom: the basal, mid, and apical posterolateral wall, followed by the apical, mid, and basal interventricular anterior septum wall; in red the segments with higher contractility.

**Two dimensional strain (2D strain)**, the most recent technique, has lately been proposed for obtaining velocities and deformation of the myocardial walls as an alternative to Doppler sampling. This method is based on the estimation of vectorial velocities instead of the analysis of the long component along the lines of the image. The algorithm identifies the vectorial velocities by 'tracking' the data obtained by the analysis of radiofrequency and black/white signals [12]. For every pixel of the image an angle-independent velocity is being estimated by selecting a pattern around the pixel examined, which is followed in the various frames, comprising the time period under examination. For those characteristics 2D strain could overcome the present limits (angle dependency and sampling in the apical segments) of the strain obtained by using the Doppler data (Figure 4).

### Reference methods

Thanks to its high temporal resolution, TDI and the methods derived from it analyze phenomena that happen in such short time periods, to elude the resolution of the human eye and could not be considered during a traditional echocardiographic evaluation [3,6]. These phenomena inherent to myocardial contractility have been addressed with methods commonly used in experimental studies, like sonomicrometric techniques, which are not reproducible in a clinical setting and for this reason cannot be defined as 'reference' methods. Consequently, the real problem consists in the lack of a definition of myocardial contractility and of a 'gold standard' that identifies it. The strain imaging technique should be used to fill this very knowledge gap.

### Technological problems

Up to now, studies conducted "in vivo" have been undertaken on a few selected patients admitted at highly specialized centers, using specific software not always widely accessible. Furthermore, companies producing ultrasound machines have not developed their products in comparable manners for reasons related to commercialization or research. As a result, at the present time, not all machines are able to produce the same parameters with the same modality. Each company was kept busy by developing its own software, then offering it prematurely and neglecting to consider whether it would turn into a commercial gadget or a truly useful tool. In addition, only recently attempts were made to standardize the nomenclature and the parameters. Presently, no agreement has been reached on the modalities of temporal analysis, on how to identify the various waves of TDI or strain curves, or on how to perform the measurements (positioning of the sample volume, size and form of the region of interest for the acquisition of the raw data). All of these issues are in part responsible for the difficulty of this method to enter "the real world".

### Contemporary applications

**Figure 5:**
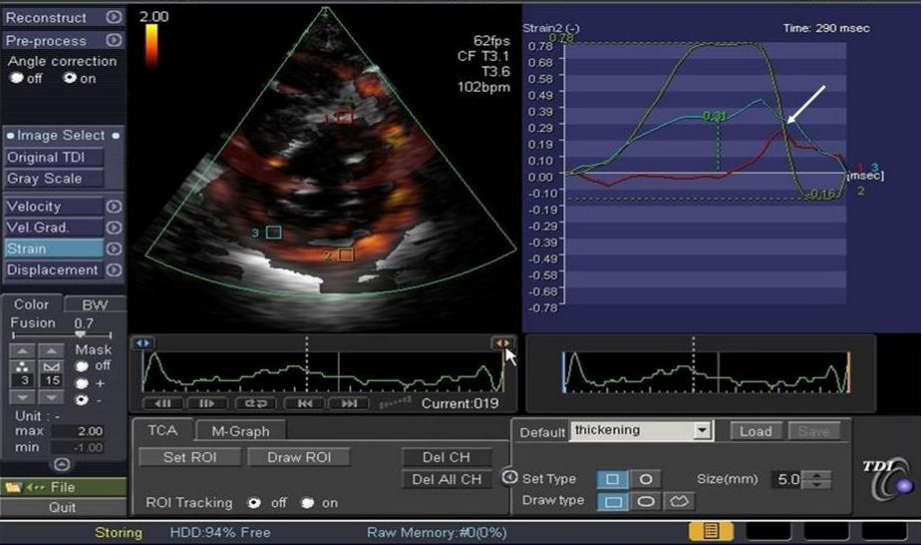
**Example of a strain curve in a patient with anterior myocardial infarction.** The green curve sampled from the normocontractile posterolateral wall shows a pronounced systolic deformation; in the red curve derived from the ischemic interventricular septum only minimal systolic myocardial deformation followed by post-systolic shortening can be seen (see arrow).

**Figure 6:**
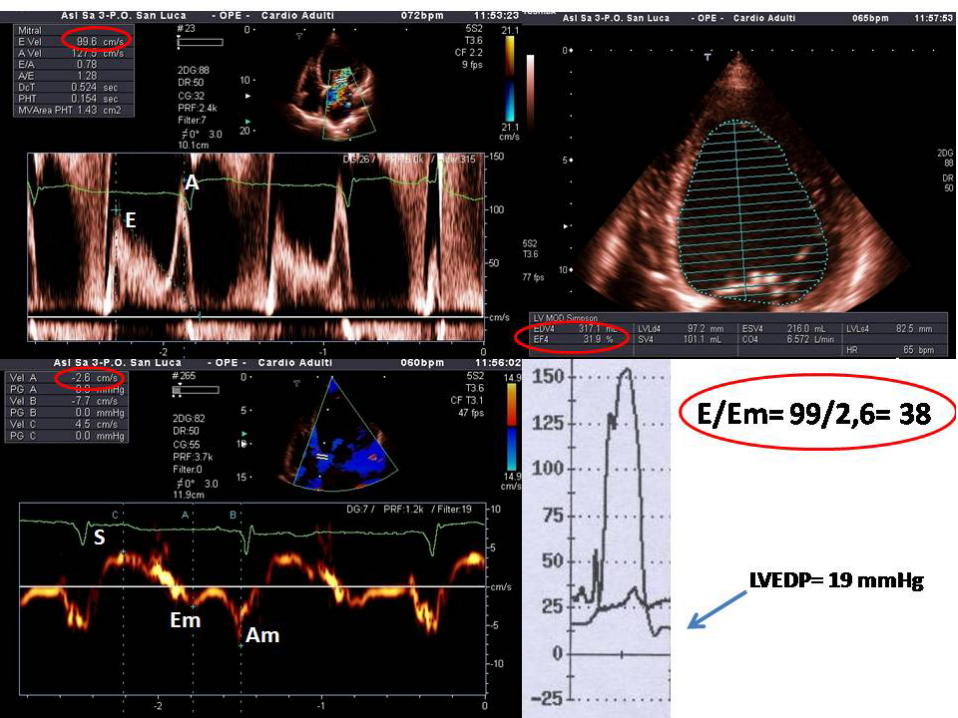
**Example of non-invasive estimation of left ventricular filling pressures in a patient with severe systolic left ventricular dysfunction (LVEF 31%; upper right panel).** Note the mitral inflow pattern consisted with abnormal relaxation (upper left panel). Only an elevated E/Em ratio > 15 allows for identification of elevated left ventricular end-diastolic pressure (LVEDP) as confirmed by invasive haemodynamic study (see arrow).

a) One very interesting application of TDI is the diagnosis of acute **myocardial ischemia**, an event that induces a change of the subendocardial fibers with prevalent longitudinal contraction, determining a reduction of peak systolic velocity, of peak strain and systolic SR (which correlate with the reduction of regional myocardial blood flow) and of the protodiastolic velocity with Em/Am inversion only after a few seconds following occlusion of the left anterior descending coronary artery [13-15]. In numerous experimental studies TDI has been shown to be able to reproduce noninvasively the typical modifications of contractile function of acute ischemia, that have already been documented in experimental studies using sonomicrometry: delayed contractile shortening followed by a late asynchronous contraction that falls in the period of isovolumetric relaxation. This last phenomenon of '*tardokinesis' *also defined as post-systolic shortening, post-systolic thickening, or post-systolic motion depending on the different technologies used to represent it, has been associated with the presence of ischemia and myocardial viability [16] (Figure 5). It also seems to have a potential role in the recognition of the genesis of ischemia in patients with left bundle branch block [17]. In addition to the observation of post-systolic motion, the pre-ejection peak velocity helps to identify myocardial ischemia [18]. TDI was proven to be feasible during physical and pharmacological stress echocardiography; however, the single multicenter study that has used TDI during dobutamine stress echocardiography, the MYDISE study, has yielded only partly encouraging results showing an advantage limited to the analysis of only a fraction of the myocardial walls and predominantly in the basal segments [19,20]. Likewise, strain and SR have been proven to be feasible during stress echocardiography, and the correct interpretation of the possible variations of the peak systolic and of the post-systolic shortening induced by dobutamine at low and high doses can represent a guide in the recognition of the different pathophysiological models of myocardial viability (stunned or hibernating myocardium), as well as after myocardial infarction of the different pathological substrates (transmural versus non-transmural necrosis) [21-23].

b) The role and value of TDI is most clearly established in the evaluation of **diastolic function**. In this context in fact the E/E_m _ratio correlates with pulmonary capillary wedge pressure and has been proven to be comparable to the plasma BNP values in the detection of elevated left ventricular filling pressures not only in critical patients admitted to intensive care units, but also in hospitalized patients independently of the value of the ejection fraction [24-27] (Figure 6). Parameters derived from the analysis of tissue velocities of the mitral ring seem to have predictive value independently and additionally to mortality after 2 years, which confers them a fundamental prognostic impact which is absolutely not negligible [26,28]. However, one must keep in mind that the preload independency of E_m _partially decreases when ejection fraction is normal, which obviously limits its use in these conditions, and that the values of E/E_m _between 8 and 15 lay in a gray zone in which it is necessary to utilize the Valsalva maneuver and to take into account the combined data of transmitral and pulmonary venous flow to ultimately be able to estimate the filling pressures [26]. The problem is that a considerable number of patients fall into this range and consequently, in these, the diagnostic contribution of TDI is diminished. This drawback is certainly compensated for in two categories of patients: those with atrial fibrillation and those after heart transplant surgery, for which, each for different reasons, the value of standard Doppler echocardiographic parameters for evaluation of diastolic function is reduced [29]. On these grounds TDI has actually entered the clinical arena for the assessment of diastolic function [26]. Beyond global diastolic function, the analysis of regional diastolic function with SR shows evidence of a reduction of the protodiastolic peak at rest that predicts with a specificity of 93% the presence of significant coronary heart disease at coronary angiography [30].

c) One of the new aspects introduced by TDI is the possibility to examine **myocardial function along the heart's longitudinal axis**. The ventricular myocardium comprises of longitudinal fibers (longitudinal contraction), which make up most of its subepicardial and subendocardial layers and the papillary muscles, and of circular fibers (radial contraction) which make up most its middle layer [31]. This spatial configuration results in the myocardium being anatomically and functionally heterogeneous. The longitudinal systolic function changes earlier in respect to radial systolic function, in ischemia as well as in myocardial hypertrophy [32,33]. Numerous clinical studies demonstrated the possibility to easily document signs of global systolic longitudinal dysfunction by analyzing the systolic velocity of the mitral ring. A peak systolic velocity S_m _> 5,4 cm predicts a left ventricular ejection fraction of 50% with a sensitivity of 88% and a specificity of 97% [34]. Peak and time to peak systolic velocity measured in the posterior wall correlate with LVEF and with the invasively measured dP/dt, in normals as well as in patients with heart disease [35]. A reduced S_m _has been reported to correlate with LVEF in patients with dilated cardiomyopathy [36]. It is important to emphasize the following aspects:

- Systolic velocity can be reduced in those parts of the mitral ring corresponding to walls with regional wall motion abnormalities. In those cases it is preferable to calculate a medium of the values obtained in at least 4 regions of the mitral ring;

- The indices of longitudinal left ventricular dysfunction do not correlate linearly with the conventional indices like LVEF, because they depend on other factors such as ventricular volumes, wall thickness and cardiac rhythm;

- The analysis of the mitral annulus is affected by the anatomical and functional status of the left atrium as well as by the annulus itself (calcification, valvular prosthesis etc...);

- Even in the presence of a normal LVEF the longitudinal systolic function decreases with age.

d) One field of application, which in itself would guarantee the survival of TDI is the study of ventricular **contractile dyssynchrony**. The development of ultrasound platforms capable of imaging at high frame rates leads to temporal resolution high enough as to allow for a meticulous analysis of the different phases of the cardiac cycle. The measurement of TDI-derived time intervals has been more easily accepted, firstly because they were related to parameters cardiologists are already confident with, secondly because they were validated in hemodynamic laboratories and thus have been confronted to a "gold standard" [37]. The application of these methods has brought upon the definition and the calculation of delays in global (interventricular) and regional (intraventricular) mechanical contraction [38]. All this has had the merit of drawing attention to the impact of dyssynchrony on cardiac performance and has shed light on the presence of significant ventricular asynchronicity even in the absence of left bundle branch block in patients with normal or near normal duration of QRS, as well as in patients with heart failure and without severely reduced LVEF [38]. The deciding driving force was a fortunate historical coincidence: the concurrent interest of the scientific community in cardiac resynchronization therapy in patients with severe heart failure despite optimal medical therapy and enlargement of the QRS complex in the electrocardiogram [39]. Evidence emerged in literature emphasizing the possible superiority of TDI in respect to ECG and traditional echocardiography in the identification of a significant number of cases of mechanical ventricular dyssynchrony which is fundamental to identify those candidate patients for implantation of a biventricular pace-maker who will be "responders" and benefit from this procedure [38,40-48]. Further, it is possible to map the delays, knowing which of the walls are being activated with greater delay and among those more capable of recovery, and thus providing important information to the electrophysiologist to plan the modality and the site of stimulation [38]. Even in this field, where it performed with great success, TDI remained a prisoner of excessive differences in methodologies in use and modalities of measurement of ventricular delays. Some authors use spectral pulsed Doppler [40], others color TDI [41,42] others strain Doppler [43,44], and others again 2D strain [45]; measuring the delays from the beginning of the QRS respectively to the beginning or the peak of the S wave; some have taken into account the absolute values, others calculated mean values, the standard deviation; emphasis was laid by some on interventricular dyssynchrony, by others on intraventricular dyssnchrony, and still by others on the sum of both combined [38,40-49] (table 1). Although two indices, those proposed by Bax et al. [41] and by Yu et al. [42,46], seem to have found broad consensus, in reality even these have failed when tested in the PROSPECT trial, a multicenter study, which gave disappointing results in the prediction of responders to resynchronization therapy [47]. The reasons for these dissatisfying results may lie in a lack of standardization in regards to the modalities of examination and of analysis. Measuring mechanical dyssynchrony is an opportunity which these new echocardiographic methods should not fail to take advantage of, because of their unique property to represent the non invasive "interpretation key" to myocardial function. The necessity for adequate training aimed at unifying the interpretation of TDI and strain curves to reduce interobserver variability is evident [47-49].

**Table 1:** Indices of ventricular dyssynchrony obtained by tissue Doppler and strain imaging

**Method**	**Parameter**	**Cut-off**
TVI	Difference between peak systolic velocities of opposite walls [41]	> 65 ms
PW- TDI	Sum of interventricular and intraventricular asynchrony [40]	> 167 ms
TVI	Systolic time-to-peak velocity -SD [42]	> 32.6 ms
TSI	Systolic time-to-peak velocity -SD [46]	> 34.4 ms
Strain Doppler	Time-to-peak of negative strain (systolic or post-systolic) -SD [43]	> 60 ms
Strain Doppler	Sum of times-to-peak of post-systolic shortening [44]	> 760 ms
2D strain	Difference of time-to-peak of radial systolic strain [45]	= 130 ms

SD = standard deviation; PW-TDI = pulsed-wave tissue Doppler; TSI = tissue synchronization imaging; TVI = myocardial velocity curves obtained with color tissue Doppler; 2D Strain = two-dimensional strain (speckle-tracking). Modified from Galderisi M et al [49].

### Future applications

a) It is undeniable that the potential development of TDI indices correlated to **right ventricular function **is being closely followed. This is because of the intrinsic limitations of standard echocardiography which does not allow for an examination of a heart chamber with such complex anatomy as that of the right ventricle. The problem becomes more evident in some patient categories, such as obese patients, those with chronic bronchopneumopathy or those patients in intensive care, for which for various reasons the acoustic window is suboptimal. In these cases it would be useful to have on hand "objective" parameters relatively independent of image quality. PW tissue Doppler seems to have this potential more than other methods; in fact a direct relationship between isovolumetric relaxation time and systolic pulmonic arterial pressure has been observed. However, in this case broader studies are needed to verify which indices can be used and in which patient categories [50-54]. In experimental studies, myocardial acceleration during isovolumetric contraction has been shown to be a load-independent parameter that correlates with telesystolic elasticity of the right ventricle, and if this is verified in clinical practice, it could become a useful and interesting parameter to measure right ventricular systolic function [55].

b) TDI and strain Doppler can catch preclinical signs of prevalently **diastolic myocardial dyfunction **in various cardiac pathologies, like hypertensive cardiopathy [56], diabetic cardiomyopathy [57], and secondary cardiopathies caused by neurological diseases like Friedreich's ataxy [6]. The distinction between pathological and physiological myocardial hypertrophy is of considerable clinical relevance, as well as the early identification of patients genetically predisposed to hypertrophic cardiomyopathy before the appearance of the very hypertrophy itself [58]. The use of TDI permits to reveal signs of early systolic dysfunction, present when conventional indices of systolic function are still normal, as in diverse conditions like amyloidosis [50], thalassemia major [60], Fabry cardiomyopathy [61], Chagas disease [62] and other rare pathologies. Besides, in Fabry's disease an improvement of peak systolic strain and SR after enzymatic substitution therapy has been documented [63].

c) Additionally, TDI and strain methods have been applied in the study of **atrial mechanical function**. Strain indices have been shown to be capable of identifying those patients with higher probability to maintain sinus rhythm after electrical cardioversion of atrial fibrillation [64]. These data seem promising because they can be applied to a wide population of patients with cardiopathies, a population with atrial fibrillation, taking in consideration recurring relapses (administration of antiarrythimic drugs and duration of therapy, anticoagulants).

### What will the future echocardiography laboratory look like?

For the purpose of establishing the measuring parameters and the best modalities of acquisition, it will be necessary on the one hand to plan prospective trials directed at testing the feasibility and reproducibility of specific protocols in defined clinical settings and on the other hand to confront these echocardiographic parameters with other methods (like nuclear magnetic resonance) capable of analyzing the same phenomena with different physical principles and modalities [14]. This kind of effort will be fundamental to increase the type and number of clinical conditions in which these methods can pass from potentially useful to certainly useful (Tab. 2), accomplishing the leap in quality from being an "industrial gadget" to being a validated diagnostic "tool". In the meantime it would be desirable for cardiologists to be open minded towards possible clinical applications of the "new technologies" of echocardiography. Echocardiography gained its renown by becoming the "battle horse" of the clinical cardiologist thanks to its widespread availability (favored by portable echo devices) and most of all by the immediateness of the morphological and functional information, which enables the experienced echocardiographer to make diagnostic judgments and prognostic predictions within minutes. To apply all this with optimal efficacy it will be necessary to structure the echocardiography laboratory in two distinctive procedural phases: A first phase dedicated to the mere acquisition of the images (possibly performed by echocardiography technicians or sonographers), and a second phase which does not require the patient's presence during which the images are analyzed to obtain all the necessary information, utilizing specialized software. Tedious off-line analysis, however, could lead cardiologists staying long periods of time in the "digital" echo lab with a corresponding drastic extension of time dedicated to each exam and the inevitable impairment of the productivity of medical doctors and their laboratories. It is therefore necessary, for the companies to focus their efforts on developing software capable of improving the definition of spectral curves linked to semi-automated analysis systems so as to render the acquisition and interpretation of the data rapid and reliable. A faster application time of the method would render it more "user friendly" promoting didactics and its definite implementation. Finally, as the Americas are no longer known as the "new world", the time will come when tissue Doppler and strain technologies will rid themselves of the limiting label "new technologies" to become familiar "partners" in our everyday cardiological diagnostic activity.

**Table 2:** Clinical applications of tissue Doppler and strain

**Parameters**	**Information**	**Clinical condition**	**TDI**	**Strain**	**SR**
Basal systolic peak	Global longitudinal systolic function	Aspecific	**-**	**-**	**+/-**
Systolic time-to-peak	Mechanical dyssynchronicity	Cardiomyopathy	**+**	**+/-**	**-**
Systolic peak	Right ventricular systolic function	Heart failure	**-**	**+/-**	**+/-**
Systolic peak and/or protodiastolic peak	Regional systolic function at rest	Ischemic Cardiopathy	**-**	**+/-**	**+/-**
Systolic peak and/or protodiastolic peak	Regional systolic function during stress	Ischemic Cardiopathy	**-**	**+/-**	**+/-**
Systolic peak	Preclinical cardiac involvement	Infiltrative Cardiomyopathy	**-**	**-**	**+/-**
Systolic peak	Monitoring of therapeutic effect	Infiltrative Cardiomyopathy	**-**	**+/-**	**+/-**
Protodiastolic peak	Ventricular filling pressures	Aspecific	**++**	**-**	**+/-**
Protodiastolic peak	Prognostic indicator	Arterial Hypertension	**+**	**-**	**-**
Protodiastolic peak	Distinction between constriction and restriction	Heart failure	**+**	**-**	**+/-**
Protodiastolic peak	Identification of pathological hypertrophy	Ventricular Hypertrophy	**+**	**-**	**+/-**
Atrial systolic peak	Prediction of response to cardioversion	Atrial Fibrillation	**-**	**+**	**-**

**SR **= Strain rate; **- **= not useful; **+/- **= useful but limited reproducibility; **+ **= potentially useful; **++ **= proven to be useful.

## Competing interests

The authors declare that they have no competing interests.

## Authors' contributions

RC wrote the paper, EB drafted the manuscript, BK translated into English, GG participated in designe of the article, AS wrote and reviewed the article and participated in its designe. All authors read and approved the final manuscript.
